# Supplementary material for: On the relation of gene essentiality to intron structure: a computational and deep learning approach
Source: Life Sci Alliance. 2021 Apr 27;4(6):e202000951. doi: 10.26508/lsa.202000951 (PMC8127325; doi:10.26508/lsa.202000951)
Supplement: Reviewer comments [file LSA-2020-00951_review_history.pdf]

Life Science Alliance

On the Relation of Gene Essentiality to Intron Structure: A Computational and Deep Learning Approach

Edward Vendrow, Joshua Vendrow, Elan Schonfeld and Ethan Schonfeld
DOI: https://doi.org/10.26508/lsa.202000951

Corresponding author(s): Ethan Schonfeld (Stanford University)

Review Timeline:

Submission Date:	2020-11-01
Editorial Decision:	2021-04-08
Revision Received:	2021-04-12
Accepted:	2021-04-15

Scientific Editor: Shachi Bhatt

Transaction Report:

(Note: With the exception of the correction of typographical or spelling errors that could be a source of ambiguity, letters and reports are not edited. The original formatting of letters and referee reports may not be reflected in this compilation.)

No full Peer Review Process File is available with this article, as the authors have chosen not to make the review process public in this case.

April 8, 2021

RE: Life Science Alliance Manuscript #LSA-2020-00951-T

Ethan Schonfeld
Stanford University

Dear Dr. Schonfeld,

Thank you for submitting your manuscript entitled "On the Relation of Gene Essentiality to Intron Structure: A Computational and Deep Learning Approach". We would be happy to publish your paper in Life Science Alliance pending final revisions necessary to meet our formatting guidelines.

We apologize for this extended and unusual delay in getting back to you. It took us an unusually long time to find a panel of reviewers for this study, and unfortunately, we were unable to retrieve comments from two of the reviewers despite multiple chasers. However, we do have comments from one of the reviewers, who is very enthusiastic about the manuscript. We also requested one of our board members, who is an expert in this field to assess the manuscript internally, and I am pleased to let you know that the board member is also satisfied with the study.

Given the positive outlook of the reviewer and the expert on our board, we would like to invite you to publish this study with us pending minor revisions that address the reviewers' points and formatting edits to comply with Life Science Alliance's guidelines.

Along with the points listed below, please also attend to the following,

-please consult our manuscript preparation guidelines https://www.life-science-alliance.org/manuscript-prep and make sure your manuscript sections are in the correct order
-please upload your main figures as single files
-please use capital letters in legends for figures, and also in manuscript text a-A
-please add your main figure legends to the main manuscript text after the references section
-please add a Category, Summary Blurb/Alternate Abstract for your manuscript in our system
-please add an Author Contributions section to your main manuscript text and insert this info in our system as well
-please add callouts for all Figures to your main manuscript text (Fig. 1A-C; Fig. 2A-C; Fig. 3A-D)
-please upload your main manuscript text as an editable doc file

If you are planning a press release on your work, please inform us immediately to allow informing our production team and scheduling a release date.

To upload the final version of your manuscript, please log in to your account:
https://lsa.msubmit.net/cgi-bin/main.plex
You will be guided to complete the submission of your revised manuscript and to fill in all necessary information. Please get in touch in case you do not know or remember your login name.

To avoid unnecessary delays in the acceptance and publication of your paper, please read the following information carefully.

A. FINAL FILES:

These items are required for acceptance.

-- An editable version of the final text (.DOC or .DOCX) is needed for copyediting (no PDFs).

-- High-resolution figure, supplementary figure and video files uploaded as individual files: See our detailed guidelines for preparing your production-ready images, https://www.life-science-alliance.org/authors

-- Summary blurb (enter in submission system): A short text summarizing in a single sentence the study (max. 200 characters including spaces). This text is used in conjunction with the titles of papers, hence should be informative and complementary to the title. It should describe the context and significance of the findings for a general readership; it should be written in the present tense and refer to the work in the third person. Author names should not be mentioned.

B. MANUSCRIPT ORGANIZATION AND FORMATTING:

Full guidelines are available on our Instructions for Authors page, https://www.life-science-alliance.org/authors

We encourage our authors to provide original source data, particularly uncropped/-processed electrophoretic blots and spreadsheets for the main figures of the manuscript. If you would like to add source data, we would welcome one PDF/Excel-file per figure for this information. These files will be linked online as supplementary "Source Data" files.

Submission of a paper that does not conform to Life Science Alliance guidelines will delay the acceptance of your manuscript.

It is Life Science Alliance policy that if requested, original data images must be made available to the editors. Failure to provide original images upon request will result in unavoidable delays in publication. Please ensure that you have access to all original data images prior to final submission.

The license to publish form must be signed before your manuscript can be sent to production. A link to the electronic license to publish form will be sent to the corresponding author only. Please take a moment to check your funder requirements.

Reviews, decision letters, and point-by-point responses associated with peer-review at Life Science Alliance will be published online, alongside the manuscript. If you do want to opt out of having the reviewer reports and your point-by-point responses displayed, please let us know immediately.

Thank you for your attention to these final processing requirements. Please revise and format the manuscript and upload materials within 7 days.

Thank you for this interesting contribution, we look forward to publishing your paper in Life Science Alliance.

Sincerely,

Shachi Bhatt, Ph.D.
Executive Editor
Life Science Alliance
http://www.lsajournal.org
Tweet @SciBhatt @LSAjournal

April 15, 2021

RE: Life Science Alliance Manuscript #LSA-2020-00951-TR

Ethan Schonfeld

Stanford University

Dear Dr. Schonfeld,

Thank you for submitting your Research Article entitled "On the Relation of Gene Essentiality to Intron Structure: A Computational and Deep Learning Approach". It is a pleasure to let you know that your manuscript is now accepted for publication in Life Science Alliance. Congratulations on this interesting work.

The final published version of your manuscript will be deposited by us to PubMed Central upon online publication.

Your manuscript will now progress through copyediting and proofing. It is journal policy that authors provide original data upon request.

Reviews, decision letters, and point-by-point responses associated with peer-review at Life Science Alliance will be published online, alongside the manuscript. If you do want to opt out of having the reviewer reports and your point-by-point responses displayed, please let us know immediately.

IMPORTANT: If you will be unreachable at any time, please provide us with the email address of an alternate author. Failure to respond to routine queries may lead to unavoidable delays in publication.

Scheduling details will be available from our production department. You will receive proofs shortly before the publication date. Only essential corrections can be made at the proof stage so if there are any minor final changes you wish to make to the manuscript, please let the journal office know now.

DISTRIBUTION OF MATERIALS:

Authors are required to distribute freely any materials used in experiments published in Life Science Alliance. Authors are encouraged to deposit materials used in their studies to the appropriate repositories for distribution to researchers.

You can contact the journal office with any questions, contact@life-science-alliance.org

Again, congratulations on a very nice paper. I hope you found the review process to be constructive and are pleased with how the manuscript was handled editorially. We look forward to future exciting submissions from your lab.

Sincerely,

Shachi Bhatt, Ph.D.

Executive Editor

Life Science Alliance

http://www.lsajournal.org

Tweet @SciBhatt @LSAjournal
